# Plasmon Enhanced Fluorescence and Raman Scattering by [Au-Ag Alloy NP Cluster]@SiO_2_ Core-Shell Nanostructure

**DOI:** 10.3389/fchem.2019.00647

**Published:** 2019-09-24

**Authors:** Chengyun Zhang, Tingting Zhang, Zhenglong Zhang, Hairong Zheng

**Affiliations:** School of Physics and Information Technology, Shaanxi Normal University, Xi'an, China

**Keywords:** plasmon enhanced spectroscopy, raman scattering, fluorescence, core-shell structure, Au-Ag alloy NP cluster

## Abstract

Silica-shell coated noble metal nanoparticles have shown a good performance in surface enhanced fluorescence and Raman scattering. However, silica-shell coated single noble nanoparticle cannot effectively enhance the optical signal due to the relative weak near-field enhancement. In this paper, [Au-Ag alloy NP cluster]@SiO_2_ core-shell nanostructure is employed to achieve the effective electric field enhancement. With the specific structure, simultaneous Raman scattering and fluorescence emission enhancement is obtained, and the enhancement comparison of fluorescence emission with Raman scattering in different type agglomeration of metal NPs is investigated *in-situ*. With different thickness of SiO_2_ shell, the optimized Raman and fluorescence enhancement systems are obtained, respectively, and corresponding study of power dependence are investigated in detail. The selectively enhanced Raman and fluorescence can be realized via controlling the shell thickness and laser power. Our work provides a non-polarization dependent [metal NP cluster]@SiO_2_ system, which may have a promising application in portable chemical and biochemistry detecting.

## Introduction

Nobel metal based nanoparticles (NPs), such as silver or gold NPs, have shown a great potential in plasmon sensor (Homola et al., 1999; Chen and Ming, 2012; Rosman et al., 2013), catalyst (Christopher et al., 2011; Han et al., 2015; Vadai et al., 2018; Zhang et al., 2018), biotherapy (Wang et al., 2014), and signal enhancement (Li et al., 2010; Ando et al., 2011; Wang and Kong, 2015) because of the abundant tunable surface plasmon resonance (SPR) peak in wide-range spectrum. The position of the SPR peak can be easily controlled through changing the morphology and material of the NPs (Liu et al., 2017). However, for pure Ag or Au NPs, it is hard to manipulate the SPR wavelength, especially at the wavelength of 400–530 nm (Liu et al., 2011; Li et al., 2017), which limits its application to some extent. Bimetallic Au-Ag alloy NPs provide a solution to the fault of the SPR wavelength, which can keep the morphology and size of metal NPs unchanged (Kuladeep et al., 2012; Rioux et al., 2014). Moreover, the Au-Ag alloy has congregated the advantages of Au and Ag, such as high chemical stability (Gao et al., 2014) and good photocatalytic properties (Tsukamoto et al., 2012; Han et al., 2016), and it is considered to be a promising candidate in metal material.

For years, many researches have been focused on plasmonic NPs enhancement of the optical signal (Ming et al., 2009; Schietinger et al., 2010; Ding et al., 2016), which is utilizing the near-field generated by the plasmonic NPs. The property of the Electricmagnetic (EM) field generated by the NPs is highly dependent on the morphology of the NPs and the features of the incident light (Sherry et al., 2005; Talley et al., 2005; Hao et al., 2007). For certain isolated NP structures, the enhancement of the near-field is highly dependent on the polarization of the incident light and the size of the NPs (Nie and Emory, 1997; Mia et al., 2019), and the effect of the enhancement is relatively lower compared with the cluster structure. For the cluster structure, the aggregation of the small particles generates lots of the hot spot, which effectively improves the intensity of the surrounding EM field (Adams et al., 2012). Furthermore, the cluster structure also has a low dependency to the polarization variation of the incident light, which simplifies the experimental condition, making it a more efficient enhancement system.

In this paper, different agglomeration types of Au-Ag alloy NPs are employed to investigate the enhancement effect of the fluorescence and Raman signal. Three different thickness levels of the SiO_2_ layer are coated on the alloy NPs and clusters to explore the optimized enhancement system for the fluorescence and Raman signal, respectively. The power dependence of the fluorescence and Raman signal are also studied on [Au-Ag alloy NP cluster]@SiO_2_ core-shell nanostructure with different shell thickness. This work offers a convenient non-polarization dependent Au-Ag alloy cluster system to enhance both the fluorescence emission and Raman scattering, which have the promising application potential in portable chemical or biochemistry detecting.

## Experiment

Sodium citrate reduction method (Lee and Meisel, 1982) are used in synthesizing citrate-reduced Au-Ag alloy NPs. First, Aqueous solution of AgNO_3_ (99.8%) and HAuCl_4_·4H_2_O, (99%) are added in proportion into boiled deionized water and keeping reaction for 10 min, then the reductant Sodium citrate (99%) is added and heating is continued for 15 min. Finally, isolated alloy NPs can be obtained after centrifugation (8,000 r/min) and washing with deionized water. We can get the alloy clusters by washing with ethyl alcohol and increasing the rpm (12,000 r/min) during a repeating washing-centrifuging cycle. It should be noted that centrifugal speed and washing solution need to be controlled in order to control the dispersion of the obtained NPs.

The modified Stöber method (Lu et al., 2002; Lessard-Viger et al., 2009) is used to coat the SiO_2_ shell on Au-Ag alloy NPs or clusters. The obtained Au-Ag alloy NPs (clusters) are dissolved in isopropyl alcohol (99.7%) and heated to 40°C. Then deionized water, ammonium hydroxide (25%) and tetraethyl orthosilicate (28.5%) are added into the colloid and keeping reaction for 3.5 h with continuous stirring. The amount of silica precursor (tetraethyl orthosilicate) is important to the thickness of the silica shell. After washing with ethanol and deionized water, the obtained core-shell structures are dispersed in deionized water and mixed with probe molecules (Rh6G). Then the mixture solution after ultrasonic agitation was left to stand in the dark for 2 days at room temperature, in order to achieve a uniformly coated fluorophore molecule on the surface of the shell. The excess molecule in the solution can be removed by washing several times.

## Results and Discussion

**Figure 1:**
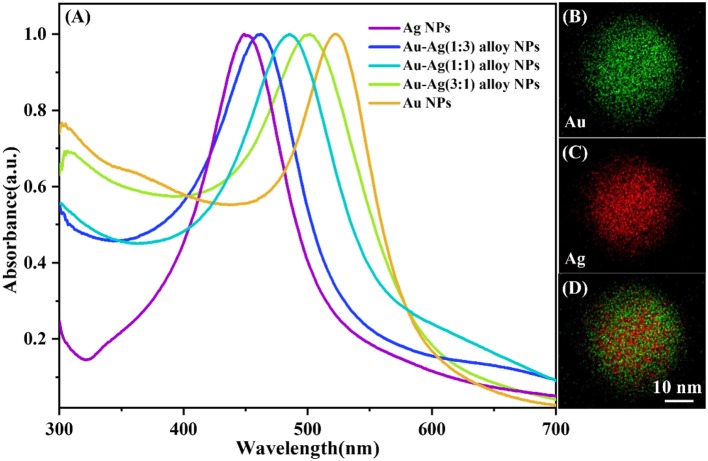
**(A)** Absorption spectra of pure Au and Ag nanospheres, and bimetallic Au-Ag alloy nanospheres with different ratio of gold to silver 1:3, 1:1, and 3:1. STEM-EDX elemental mapping image of Au-Ag alloy NP with the ratio of gold to silver 1:1, where the green represents Au **(B)**, red represents Ag **(C)**, and the merged image **(D)** from single Au-Ag alloy nanoparticle.

**Figure 2:**
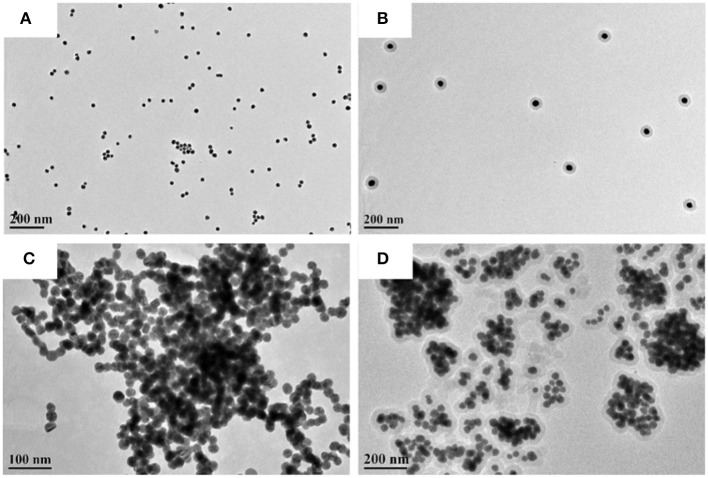
TEM images of **(A)** isolated Au-Ag alloy NPs. **(B)** [isolated Au-Ag alloy NP]@SiO_2_. **(C)** Au-Ag alloy NP cluster. **(D)** [Au-Ag alloy NP cluster]@SiO_2_.

With different ratios of the Au and Ag content, the SPR peak can be easily tuned from 400 to 530 nm (Figure 1A). The 1:1 ratio of the Au and Ag is chosen in the following parts, and corresponding elemental distribution is obtained through the energy dispersive X-ray (EDX) elemental mapping. As shown in Figures 1B–D, the elemental mapping image (Figures 1B–D) of Au-Ag alloy NPs shows that the ratio of the Au and Ag content is 1:1. Besides, the morphology of the obtained isolated Au-Ag alloy NPs, Au-Ag alloy NP clusters, [isolated Au-Ag alloy NP]@SiO_2_ and [Au-Ag alloy NP cluster]@SiO_2_ core-shell structure are characterized with TEM. As shown in Figure 2A, the uniform-sized Au-Ag alloy isolated nanospheres are synthesized with high dispersity. The thickness controllable SiO_2_ layer can be evenly coated on the surface of the Au-Ag alloy nanospheres (Figure 2B). Figure 2C shows the morphology of the Au-Ag alloy cluster which is composed with the uniform sized Au-Ag alloy nanospheres, and corresponding SiO_2_ coated structure is shown in Figure 2D. Meanwhile, the different thickness of the SiO_2_ layer can also be selectively coated on the cluster.

In order to evaluate the ability of the enhancement on Raman scattering and fluorescence emission of different system, Rh6G molecule is chosen as a fluorescence/Raman detecting molecule, which is uniformly absorbed on the surface of SiO_2_ layer. Here we choose [isolated Au-Ag alloy NP]@SiO_2_ system (Figure 2A) to compare with the [Au-Ag alloy NP cluster]@SiO_2_ system (Figure 2C). Both above systems are coated with Rh6G in same concentration. Surface enhanced Raman scattering (SERS) and surface enhanced fluorescence (SEF) spectra were investigated with quantitative core-shell particles system. The number of molecules absorbed on the surface of the SiO_2_ is different due to the different surface area. Thus, all the spectra are normalized with the surface area, and signal intensity is from the per unit area of the surface.

**Figure 3:**
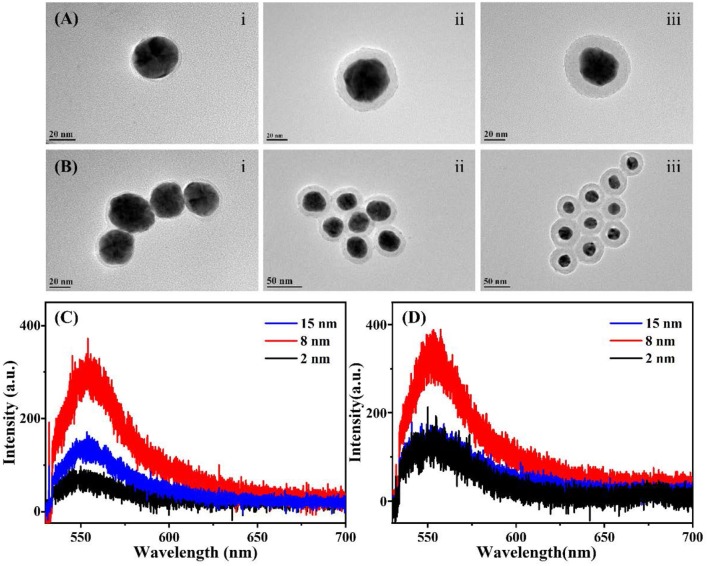
TEM images and SEF spectra of Au-Ag alloy@SiO_2_ shell isolated system with different thickness of SiO_2_ layer. **(A)** [Isolated Au-Ag alloy NP]@SiO_2_ with the SiO_2_ layer of 2 nm (i); 8 nm (ii); 15 nm (iii), respectively. **(B)** Aggregated [isolated Au-Ag alloy NP]@SiO_2_ core-shell structure with the SiO_2_ layer of 2 nm (i); 8 nm (ii); 15 nm (iii), respectively. **(C)** SEF spectra of the single [isolated Au-Ag alloy NP]@SiO_2_ core-shell NP with different SiO_2_ layer shown in **(A)**. **(D)** SEF spectra of a certain number of closely arranged core-shell NPs with different SiO_2_ layer shown in **(B)**.

Three different levels of thickness (2, 8, and 15 nm) of SiO_2_ shell are selectively chosen to investigate the sensitivity of SERS/SEF effect. SEF spectra of single [isolated Au-Ag alloy NP]@SiO_2_ (Figure 3A) and multiple tightly distributed shell isolated core-shell NPs (Figure 3B) with different shell thickness is shown in Figures 3C,D. It is obvious that the fluorescence signal is effectively enhanced in the core-shell system, especially for the 8 nm SiO_2_ coated system. However, no obvious SERS signal could be detected in both two spectra, which means that the Raman scattering cannot be effectively enhanced and observed by the above single Au-Ag alloy NPs@SiO_2_ shell isolated NP or multiple tightly distributed shell isolated core-shell NPs.

**Figure 4:**
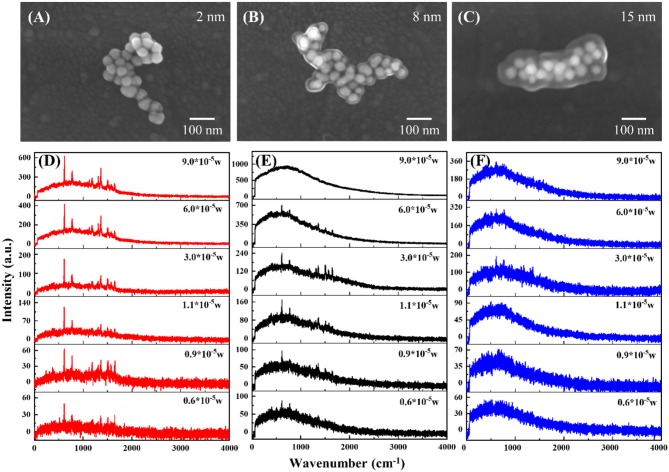
SEM images of [Au-Ag alloy NP cluster]@SiO_2_ core-shell nanostructure with the SiO_2_ layer of 2 nm **(A)**, 8nm **(B)** and 15 nm **(C)**, respectively. Raman spectra of [Au-Ag alloy NP cluster]@SiO_2_ under different laser power excitation with the SiO_2_ layer of 2 nm **(D)**, 8 nm **(E)** and 15 nm **(F)**, respectively.

Figure 4 shows the enhancement effect of Raman and fluorescence signal of the [Au-Ag alloy NP cluster]@SiO_2_ system. As shown in Figures 4A–C, three different thickness levels of the SiO_2_ layer (2, 8, and 15 nm) are evenly coated on the Au-Ag alloy cluster. Figures 4D–F show the power-dependence *in-situ* spectra of the corresponding samples shown in Figures 4A–C. When the thickness of the SiO_2_ is 2 nm (Figure 4D), both Raman and fluorescence signal are obviously enhanced by the system. As the laser power decreases, the intensity of the Raman scattering and fluorescence emission have obvious damping, but the Raman signal can still be clearly observed under the lowest power excitation, which results in the high intensity EM field generated by the alloy cluster. When comparing to the spectra of Figures 4E,F that were obtained from the cluster coated with thicker SiO_2_, the fluorescence intensity of the cluster coated with 2 nm SiO_2_ is obviously lower under the same power laser excitation, which is attributed to the quenching effect of SPR. When the molecules are located in the vicinity of the plasmonic metal NPs, the fluorescence emission intensity will be affected by many factors, such as the enhanced local field and the energy transfer between the molecule and plasmonic metal and the field. In general, the local EM field brings with it the enhancement effect of the excitation of molecules, while the effect of energy exchange between metal and molecules on SEF will change from quenching to enhancement with an increase of spacing (Zhang et al., 2016). The EM fields are confined at the surface of the shell, as its spatially inhomogeneous distribution is dramatically decayed with increased thickness of the SiO_2_, and the overall SEF results is dependent on the competition effects between the excitation enhancement and quenching effect. Alloy NP cluster coated with 8 nm SiO_2_ shows the best enhancement effect on the fluorescence of the Rh6G molecular (Figure 4E). For the optimized shell thickness (8 nm) of SEF, it is interesting that the SERS signal cannot be observed under the relative high-power excitation, due to the ultra-high enhanced fluorescence emission that may cover the SERS signal. Thus, it can be seen that the Raman scattering can only be obviously observed between the power of 0.9 × 10^5^ and 3 × 10^5^ W with the 8 nm shell. Moreover, as for the [Au-Ag alloy NP cluster]@SiO_2_ with 15 nm silica layer, because the EM enhancement is too weak at the surface of shell far away from the cluster, both the Raman and fluorescence signal cannot be effectively enhanced.

**Figure 5:**
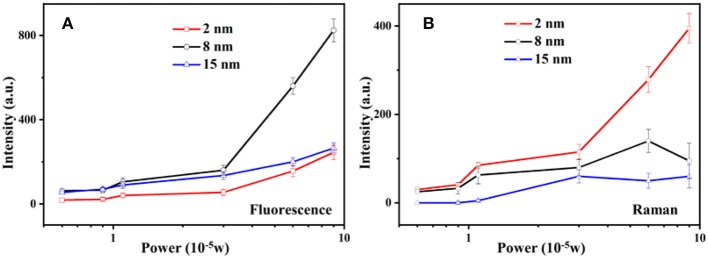
Power dependence of the intensity of SEF **(A)** and SERS **(B)** with [Au-Ag alloy NP cluster]@SiO_2_ core-shell structure.

Comparing the influence of the SiO_2_ layer to the SERS/SEF intensity under different power excitation, Raman scattering and fluorescence emission intensity of the Rh6G molecules are obtained from the per unit area of the molecules adsorbed on the surface of the SiO_2_. Figure 5 shows the relation between the fluorescence (Raman) intensity and the laser power of different thickness of SiO_2_ coated Au-Ag NP cluster. As shown in Figure 5A, due to the fluorescence quenching effect with thin shell (2 nm) and the far distance with the EM enhanced field with thicker shell (15 nm), Au-Ag alloy NP cluster coated with 8 nm silica shell is the optimized system for the fluorescence enhancement. Figure 5B indicates the relation between the Raman intensity and the laser power. Different from the fluorescence emission, the intensity of Raman scattering demonstrates a positive correlation to the intensity of the biquadrate of the EM field (∣E∣^4^). Thus, NP cluster coated with 2 nm silica shell shows the great performance in SERS. Nevertheless, in core-shell structure, to get the high quality of the SERS signal, the influence of fluorescence has to be excluded due to the fluorescence signal coverage to the Raman signal. The alloy NP cluster coated with 8 nm shows a great performance in fluorescence enhancement, which means it is unsuitable for the SERS enhancement system. The anticlimactic intensity of the Raman scattering at the largest power excitation, shown as the black line, results in the competition between the fluorescence and Raman signal.

## Conclusion

Isolated Au-Ag alloy NPs and Au-Ag alloy NP clusters are employed to investigate and compare the enhancement effect of the fluorescence and Raman signal. Rh6G molecular with the same concentration are absorbed on three different thickness levels of the SiO_2_ layer which is coated on the Au-Ag alloy NPs and clusters. Under the same power laser excitation, the single [isolated Au-Ag alloy NP]@SiO_2_ particle and their aggregates cannot effectively enhance the Raman scattering of the adsorbed molecular. However, as seen from the strong near-field enhancement of the alloy NP cluster, both 2 and 8 nm SiO_2_ coated cluster system can obviously enhance the Raman signal, and Au-Ag alloy NP cluster with 8 nm silica shell shows the best performance in fluorescence enhancement. This work offers a convenient non-polarization dependent Au-Ag alloy NP cluster system to enhance both the fluorescence and Raman signal, which have promising application potentials in portable chemical or biochemical detection.

## Data Availability Statement

All datasets generated for this study are included in the manuscript/supplementary files.

## Author Contributions

CZ and HZ conceived the idea. CZ did the experiment and the measurement. CZ and TZ discussed and analyzed the results. TZ and CZ wrote the paper. All the authors revised the paper.

### Conflict of Interest

The authors declare that the research was conducted in the absence of any commercial or financial relationships that could be construed as a potential conflict of interest.
